# Do targeted intergovernmental fiscal transfers improve health outcomes? Evidence from Kenyan decentralization using the difference-in-differences technique

**DOI:** 10.1186/s12961-024-01272-x

**Published:** 2024-12-20

**Authors:** Bishnu Bahadur Thapa, Momotazur Rahman, Lawrence Were, Richard Wamai, Omar Galárraga

**Affiliations:** 1 Department of Health Services, Policy and Practice, School of Public Health, Brown University, Providence, United States of America; 2 Department of Health Sciences, School of Public Health, Boston University, Boston, United States of America; 3 College of Social Sciences and Humanities, Northeastern University, Boston, United States of America

**Keywords:** Intergovernmental fiscal transfer, Decentralization, Devolution, Health outcomes

## Abstract

**Background:**

As envisioned by the 2010 Constitution, Kenya implemented a devolved system of government in March 2013, setting up 47 counties and a corresponding number of local governments. These counties differed in their levels of development. While counties such as Nairobi and Kiambu led in social and economic indicators, others such as Turkana, Mandera and Wajir were at the bottom of the list. Keeping the between-country disparities and the need to remedy those disparities in mind, the national government used formula-based criteria to determine counties’ eligibility for the receipt of financial resources. On the basis of these criteria, counties were classified into marginalized and nonmarginalized counties. The marginalized counties were the 14 (of the 47) most socially and economically disadvantaged counties. These counties receive additional financial resources, which we call targeted intergovernmental fiscal transfers (i.e. fiscal transfers from the national government to county governments).

**Methods:**

We used the difference-in-differences (DID) technique and fixed effects models to estimate the effects of these targeted intergovernmental fiscal transfers on human immunodeficiency virus (HIV) incidence and diarrhoea incidence.

**Results:**

The results revealed that the counties receiving those transfers experienced a statistically significant decline in the incidence of diarrhoea but had no impact on the incidence of HIV. Our study fills a major gap in causal evidence linking intergovernmental fiscal transfers to health outcomes, especially in the context of low–middle-income countries in a newly decentralized setting.

**Conclusions:**

Our results imply that targeted intergovernmental fiscal transfers may be effective at improving some subnational health outcomes, and therefore in reducing within-country health inequalities.

**Supplementary Information:**

The online version contains supplementary material available at 10.1186/s12961-024-01272-x.

## Introduction

### Background

Intergovernmental fiscal transfers (IGFTs) refer to the financial resources transferred from the national (or central) government to subnational governments. They are commonly used by countries to achieve decentralization objectives. Central to the decentralization objectives is the idea that the centre of power and resources must be as close to the people as possible and that the people should have opportunities to participate in matters that matter to them the most because they know what is best for them [1].

IGFTs primarily address two types of fiscal imbalances: vertical and horizontal. Vertical fiscal imbalance occurs when there is a resource difference between the national government and the subnational governments. The difference occurs because the national governments, owing to their revenue-raising authority, possess resources, whereas the subnational governments undertake the bulk of public service delivery and therefore spend resources. Horizontal imbalance occurs because within a given country, subnational governments can (and often do) differ in their ability to raise revenues and finance public services such as health and education [2, 3].

Existing studies suggest that in decentralized countries, a vast majority of public sector spending, including healthcare spending, occurs at the subnational level (see Supplementary Material Table A1) [4]. Given the magnitude of subnational health spending, understanding the extent to which these transfers may translate into positive health outcomes and how those outcomes vary across different subnational governments is important.

However, evidence on whether and how IGFTs impact subnational health outcomes in low- and middle-income countries (LMICs) is limited [5]. This study contributes to the literature at the intersection of IGFTs and health by adding to the body of work examining the causal effects of IGFTs on health outcomes. This is important because causal evidence in this space is very scant, especially in the context of LMICs. Part of the reason for this scarcity is that IGFTs are endogenous to subnational-level public spending and that spending is endogenous to outcomes. In the absence of good natural experiments, disentangling the effects of IGFTs on health outcomes (or other related outcomes of interest) is a challenge [6]. Our paper addresses this by using the difference-in-differences (DID) technique, a rigorous quasi-experimental technique, to evaluate the impacts of targeted IGFTs in Kenya.

Second, this study holds relevance to the agenda of financing for universal health coverage (UHC) [7]. Because IGFTs are a source of revenues and therefore a key driver of fiscal space for health at the subnational level, they play a crucial role in addressing within-country disparities in health outcomes [8]. Finally, from a policy perspective, this study is important because of the types of outcomes studied. Existing studies undertaken in the context of decentralization in LMICs have focussed largely on mortality outcomes, such as infant mortality [9, 10], neonatal mortality [10], immunization [11, 12] and other outcomes. Within the context of decentralization in LMICs, no studies that we know of have specifically examined human immunodeficiency virus (HIV) and diarrhoea incidence. By focussing on HIV and diarrhoea outcomes, our study directly informs Kenya’s priority health policy agenda, especially since HIV/acquired immunodeficiency syndrome (AIDS) and diarrhoea continue to be two of the three major killers in Kenya [13].

### Kenyan context

Kenya decentralized in early 2013 following the mandate of the 2010 constitution. As part of long-running health system reforms to improve health, from the mid-1980s, the district administrative structure had envisioned a devolved system of health services, which was formally outlined in the government’s health policy framework of 1994 [14, 15]. The constitutional mandate was designed to address continuing inequities caused by corruption, patronage, tribalism and other socio-economic issues that have afflicted the country for several decades [16, 17]. With constitutional decentralization, Kenya created 47 county (local) governments with significant political, fiscal and administrative responsibilities, including making service delivery decisions related especially to health and early childhood education. Article 203(2) of the Constitution envisions that “not less than fifteen per cent” of national government revenues would be allocated to counties [18].

However, the newly created counties differed in their level of socio-economic and health development, population and geographical size. While counties such as Nairobi and Kiambu led in social and economic indicators, others such as Turkana, Mandera and Wajir were at the bottom of the list [19]. Keeping the between-country disparities and the need to remedy those disparities in mind, as stipulated in Sect. 203 of the Constitution, a formula-based criterion was used to designate counties as marginalized and nonmarginalized [20]. Marginalized counties were the 14 most socio-economically disadvantaged counties, whereas the remaining 33 counties were nonmarginalized counties. Stipulated in the classification system, marginalized individuals receive additional financial resources annually for improved public service provision, including healthcare [21–23].

**Fig. 1:**
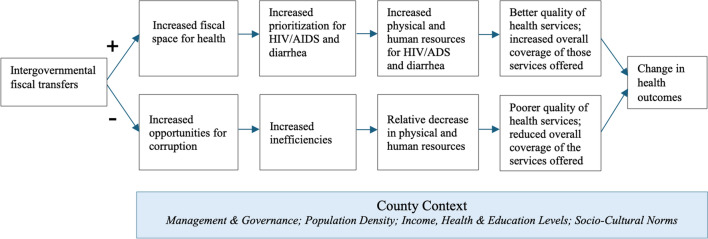
Stylized conceptual framework

We hypothesized that additional financial resources would likely lead to improved health outcomes for the marginalized countries. First, the additional resources directly translate into increased fiscal space for health. An increase in financial resources increases the size of the overall “resource pie” for the marginalized counties, and this in turn increases resources for health (as well as other sectors). Increased resources for health should then translate into increased resources for HIV/AIDS and diarrhoea programming. Second, because HIV/AIDS and diarrhoea are the two priority diseases for Kenya, marginalized counties may choose to spend a relatively greater share of their additional resources on HIV/AIDS and diarrhoea compared with other priorities in health. In both these scenarios, because marginalized counties end up devoting more resources to address HIV/AIDS and diarrhoea, we hypothesized that the marginalized counties would see a decline in both HIV incidence and diarrhoea incidence. A priori, there is no guarantee that additional resources translate into an improvement in health outcomes. In fact, literature on decentralization theorizes that such additional resources can increase opportunities for corruption [24]. Similarly, if the increase in financial resources is not accompanied by a concomitant increase in skilled manpower to manage those resources, inefficiencies may emerge. Increased corruption and/or increased inefficiency may in turn lead to poorer health outcomes at the county-level [24]. A stylized conceptual framework is presented as Fig. 1 below.

## Methods

### Data

We used data from the Institute of Health Metrics and Evaluation (IHME). The data span a total of 14 years between 2006 and 2019 and contain county-level information on outcomes as well as other socio-economic variables. We used 7 years of data prior to the implementation of decentralization and 7 years of data after decentralization began. This translated into a total of 658 county-years. As with many other data available through IHME, these data are model-based and were produced as a part of the 2019 Global Burden of Disease study [25]. For county-level analysis in Kenya, these are the best data available for public use.

To augment our main analysis, we also used official data from the Office of the Controller of Budget of the Government of Kenya. We extracted data from publicly available county-specific annual reports on budget implementation for 2013–2014 through 2018–2019 [26–31].

### Variables

Our outcomes for the main analysis were HIV incidence and diarrhoea incidence, both of which were measured as rates and expressed as the number of new cases per 100,000 people. For sensitivity analysis, we also examined the prevalence of HIV and diarrhoea. Our main independent (exposure) variable was a binary variable denoting a county’s intervention status, with 1 referring to the intervention counties and 0 to the comparison counties. A total of 14 counties were categorized as marginalized (intervention group), and 33 were categorized as nonmarginalized (comparison group) (see Supplementary Material parts B, C and D for relevant details).

### Empirical strategy

We used the difference-in-differences (DID) technique to estimate the causal effects of the intervention on HIV incidence and diarrhoea incidence. We fit a panel data-based linear fixed effects model [equivalent to a two-way fixed effects (TWFE) model] to estimate the effects of the intervention. In the presence of single intervention timing (rather than staggered timing), the TWFE model correctly estimates the average treatment effects in a difference-in-differences setting [32]. The DID estimation technique relies on the parallel trends assumption. This assumption implies that the average change in the outcome variable in the comparison group represents the counterfactual change in the outcome measure in the intervention group in the absence of the intervention [33]. Therefore, we first graphically tested the parallel trends assumption (the results are provided in Supplementary Material part F, Figs. A1 and A2). Then, for each outcome, we ran unadjusted and adjusted regressions following the canonical DID approach, which assumes two periods (pre and post) for the two groups [32]. An illustration of the canonical DID model is provided in Supplementary Material part F.

Our adjusted models controlled for income, education, share of the urban population, per capita health spending, percentage of antenatal care (ANC) coverage and percentage of children immunized with diphtheria tetanus toxoid and pertussis (DTP3) vaccination. Income was defined in terms of gross domestic product (GDP) per capita (with 2010 serving as the base year), and education was proxied using maternal education (expressed in terms of the mean number of years of schooling). All our models included time (i.e. year) and unit (i.e. county) fixed effects as well as robust standard errors.

### Sensitivity analysis

As a sensitivity analysis, we applied three tests. We first fit a new more general form of the canonical DID model that included interactions between the intervention and each timepoint (i.e. year) during the study window. This approach allowed us to see explicitly how the impact of the intervention changed throughout the study period. Second, we tested to see whether the results held true when propensity score matching (PSM) was used along with DID technique [34]. Briefly, we estimated a propensity score, which was then used to create a matched control group with observable characteristics similar to the intervention group. The matching was done at the county level using one-to-many matching with replacement. The matching variables included all the controls available [i.e. income, education, share of the urban population, per capita health spending, percentage of antenatal care (ANC) coverage, and percentage of children immunized with DTP3 vaccination]. Third, we conducted a placebo test (using years from 2006 through 2012) under the hypothetical assumption that the intervention occurred in 2009 rather than 2013. Fourth, since our outcomes are expressed as incidence rates, we tested whether fitting a Poisson model changes the estimated relationship. Finally, we used the prevalence rate as the outcome measure for both HIV and diarrhoea.

## Results

Table 1 presents summary statistics for the study population at baseline. We defined the baseline as the 3-year average of the years 2010–2012, the 3 years immediately prior to the intervention year. The two groups were quite different from each other at baseline. This is to be expected because if they were not different, the intervention would likely not have occurred in the first place.

**Table 1:** Baseline summary statistics

	Comparison (*N* = 33)		Intervention (*N* = 14)	
Variable	Mean	SD	Mean	SD
GDP per capita in USD, base 2010	2451.70	818.13	1700.45	449.20
Maternal education (in years)	8.37	0.80	4.35	1.99
Proportion of urban population	0.42	0.25	0.14	0.15
Antenatal care coverage	0.61	0.09	0.52	0.12
DTP3 vaccination coverage	0.90	0.07	0.80	0.13
Health expenditure per capita in $$	118.36	36.21	64.38	23.22

*GDP *gross domestic product, *DTP3* diphtheria tetanus toxoid and pertussis (DTP3) vaccine, *SD* standard deviation, *N* counties

$$: In US dollars

Counties in the intervention group were poorer and less educated. They we are also substantially more rural, with only 14% of their population residing in urban areas (versus 42% in the comparison counties). Indicators for healthcare performance, such as antenatal coverage and DTP3 vaccination coverage, also suggest stark differences between the two sets of counties. Access to clean water, good hygiene and improved sanitation was a challenge for both sets of counties, and the comparison group counties were slightly better off.

Table 2 presents estimates from the canonical differences-in-differences (DID) model. In models 1 and 3, we examined HIV incidence as the outcome measure, whereas in models 2 and 4, we examined diarrhoea incidence.

**Table 2:** Impact of intervention on HIV incidence and diarrhoea incidence

	Unadjusted		Adjusted	
	HIV incidence (1)	Diarrhoea incidence (2)	HIV incidence (3)	Diarrhoea incidence (4)
Intervention^*^post [DID estimate]	45.55^***^	−2222.29^**^	15.97	−4878.28^***^
	(15.379)	(955.71)	(14.69)	(1193.74)
Constant	326.11^***^	107,445.65^***^	971.49^***^	98,324.58^***^
	(10.68)	(449.91)	(187.83)	(11,378.41)
County-years	658	658	658	658

Table 2 presents the impact of difference-in-differences (DID) estimates of the impact of the intervention on HIV incidence and diarrhoea incidence. The adjusted results control for income, maternal education, the proportion of the urban population, the antenatal care (ANC) coverage rate, health expenditure per capita and the DTP3 vaccination rate. All the models include year and county fixed effects; 2006–2012 is the pre-period, and 2013–2019 is the post-intervention period. The baseline (reference) year for all the models is 2012. ***p < 0.01, **p < 0.05, *p < 0.1.

Although the unadjusted results for HIV incidence are statistically significant, the adjusted results suggest that the intervention did not significantly change. For diarrhoea, the unadjusted results suggest that the intervention led to a 2.1% reduction in the incidence of diarrhoea, whereas the adjusted results suggest that the intervention led to a nearly 5% reduction in the incidence of diarrhoea. Both results are statistically significant.

Since our analysis was based on our satisfying conditional parallel trends assumption, our preferred models are adjusted models. The results from the first sensitivity analysis, which was based on the event-study design type, are consistent with these results. The results revealed that the intervention counties did not experience any change in the incidence of HIV after 2013 but experienced a reduction in diarrhoea incidence. The reduction in diarrhoea incidence began in approximately 2016, 3 years after the intervention. Results from the sensitivity analysis involving propensity score matching (PSM) in a DID setup were consistent with the main results (i.e. no change observed for HIV incidence, but a significant reduction observed for diarrhoea incidence). In fact, the magnitude of the effect was even stronger. It would be important to note here, however, that the sample size reduced (and the standard errors increased) as a result. Regression estimates based on the placebo test showed that there was no statistically significant difference between the treatment group and the control group, as expected. Other results from the sensitivity analyses aligned with the findings on the basis of the canonical DID setup. See the Supplementary Material (parts G, H, I and J for detailed results of the sensitivity analysis).

## Discussion

Our study revealed that, relative to the comparison group, the intervention group presented a statistically significant reduction in diarrhoea incidence. The diarrhoea incidence in the intervention group decreased by approximately 5%. The decline was manifested largely in later years (2017, 2018 and 2019). There was no statistically significant effect of the intervention on the incidence of HIV.

Several interrelated factors may explain the observed effects on outcomes. The first has to do with how HIV/AIDS services have been delivered in Kenya. As in other countries in Asia and Sub-Saharan Africa, HIV/AIDS service delivery has primarily operated as a vertical program in Kenya [35]. Service delivery approaches for HIV/AIDS are still being adapted to the new set of government structures at the county level.

In contrast, diarrhoea-related efforts have been largely led by the Ministry of Health and followed a more integrated public health approach involving partnerships between government and nongovernment entities. These efforts were typically embedded into government programming at all levels, central, provincial and district, even before the 2013 decentralization. The efforts have entailed working within the established government structures and following a more decentralized approach in service delivery, something that has historically not been the case with HIV/AIDS. Decentralization likely made it even easier to undertake diarrhoea programming, in part because of the reduced administrative/bureaucratic burden, as there was no longer a need to keep coordinating with several layers of government (such as provincial and central), since counties now have substantially more independence over decision-making. Counties are responsible for implementing and coordinating water, sanitation, hygiene (WASH) programs that directly impact diarrhoea [36].

The observed results could also be accounted for by county differences in budgetary prioritization. The results may reflect the possible effect of differential county spending in terms of health spending per capita as well as the proportion of overall county budgets. To better understand the composition of county-level spending, we extracted and examined data from the annual budget reports between 2013–2014 and 2018–2019 published by the Office of the Controller of Budget of the Government of Kenya [26–31]. We found that, relative to the comparison group, the intervention group spent a greater share of its funds on social sectors [i.e. including education and water, sanitation, and hygiene (WASH) combined] (see Supplementary Material part J for details). To the extent that spending reflects priorities, the marginalized counties appear to have prioritized investing in sectors that matter more for addressing diarrhoea.

Another reason why the intervention counties experienced a reduction in diarrhoea incidence might be related to the nature of the disease itself. Behavioural change communication (BCC) related to HIV is complex. It is more difficult to talk about sexual behaviours than to talk about handwashing, toilets and ways to purify water at home [37].

Although the evidence on Kenyan decentralization is still building, our findings are in line with what some of the most updated evidence suggests. In a recently completed comprehensive analysis of Kenyan decentralization, the World Bank (2022) noted that, despite the continuation of disparities in the country, service delivery outcomes for devolved sectors [such as health and early childhood development education (ECDE)] have especially improved for lagging counties [38]. The report notes that counties

“…with inferior services, which tend to be counties that are poorer and more sparsely populated, have been able to spend more per capita on service delivery than those in urban, wealthier, and more populous counties. There are early signs of reduction in disparities in some areas of health…” (page 35).

Furthermore, the report notes that the counties

“…have generally expanded and invested in services that were devolved to them. Access to, and use of, county services has increased in health and other sectors that had previously been neglected, such as rural water and ECDE” (page 37).

The findings of our study should be understood with caveats. First, we defined treatment on the basis of marginalization status announced and implemented in 2013. At that time, the intervention (or the marginalized) counties collectively were going to annually receive 0.5% of the total national revenues collected for a given year [18]. What was not made clear is the exact share each county was going to receive. We assumed for this study that each of the marginalized counties received an equal share. In reality, some counties may have received more and others less, but since we combined all the counties in a single group (the intervention group), the exact amount received by each county is less relevant for analysis. Second, the possibility that our estimates may be biased cannot be ruled out. Biases may have occurred if unobserved confounding (e.g. unobserved county-specific attributes) were driving the difference in health outcomes between the treatment and the control counties. Biases also may have arisen because of potential reverse causality. We may have incorrectly attributed the improvement in health outcomes in marginalized counties to IGFTs when in fact IGFT was designed as a response to the poor health outcomes in those marginalized counties. While the results from the propensity score matched DID model (which are consistent with the main results) provide further credence to the results, endogeneity concerns cannot be completely ruled out. Third, evidence based on popular national media and published qualitative findings suggests that there have been delays in transferring funds to countries [39]. It is likely that in any given year, eligible counties received only a portion of what they qualified for. If counties had received all the resources allocated to them, the estimated impacts could be greater. Fourth, while this study is based on the best available county-level data on Kenya, it is limited by the fact that these data are modelled. In this sense, our findings are reliable and valid to the extent that the modelled data (produced by the IHME) are reliable and valid. However, because research papers based on these data have already been published, we are reasonably assured of the data used [25]. Fifth, while the data extracted from county budget reports provided important insights into county-level spending, the data were not based on any standardized reporting formats. Different counties report data differently, even though the same counties reported data slightly differently over the 6-year period between 2013–2014 and 2018–2019. During data extraction, efforts were made to ensure that the numbers were broadly comparable.

## Conclusions

This study revealed that targeted IGFTs in Kenya led to a relative decline in the incidence of diarrhoea but had no impact on the incidence of HIV. Taken together, our results imply that targeted intergovernmental fiscal transfers can improve health outcomes. Considering the United Nation’s growing emphasis on reducing within-country inequalities in health and other dimensions of wellbeing, our findings point to the use of targeted IGFTs as a potential way to address within-country inequalities.

With an age of less than a decade, Kenyan decentralization is still in a stage of relative infancy. Our study is an attempt to understand some of the early effects of a policy intervention implemented as part of decentralization, and at best, these effects are partial. Future studies should consider examining mortality-based outcomes (for example, diarrhoea-related deaths and HIV-related deaths). Additionally, such studies should consider examining service delivery outcomes outside of health (for example, education outcomes). Such an examination can reveal the extent to which the impacts of targeted transfers are broad-based and durable. Studies of this kind can help validate and guide well-intentioned decentralization policies that aim to solve long-standing health inequalities in Kenya and similar LMICs.

## Supplementary Information

Supplementary material 1.

## Data Availability

No datasets were generated or analysed during the current study.

## Abbreviations

ANC: Antenatal care
BCC: Behavioural change communication
DID: Difference-in-differences
GDP: Gross domestic product
HIV: Human immunodeficiency virus
IGFTs: Intergovernmental fiscal transfers
IHME: Institute of health metrics and evaluation
LMICs: Low- and lower-middle-income countries
PSM: Propensity score matching
TWFE: Two-way fixed effects
UHC: Universal health coverage
WASH: Water, sanitation, hygiene
