# Supplementary material for: Do targeted intergovernmental fiscal transfers improve health outcomes? Evidence from Kenyan decentralization using the difference-in-differences technique
Source: Health Res Policy Syst. 2024 Dec 20;22:172. doi: 10.1186/s12961-024-01272-x (PMC11660824; doi:10.1186/s12961-024-01272-x)
Supplement: Supplementary file 1 — Supplementary material 1. [file 12961_2024_1272_MOESM1_ESM.docx]

**Supplementary Material**

Intergovernmental Fiscal Transfers and Health Outcomes in Kenya

**PART A: Sub-National Health Spending**

**Table A1**: Sub-national Health Spending in Low-middle-income countries

| Country | Total health expenditure per capita (current US$) | Govt. health expenditure (% of GDP) | Share of govt. spending in total health expenditure (%) | Sub-national health spending (as a % of total govt. health spending) | Year | Source for sub-national data |
| --- | --- | --- | --- | --- | --- | --- |
| Argentina | 1074 | 4.9 | 67.7 | 57 | 2004 | World Bank, 2012 |
| Brazil | 1083 | 4.7 | 48.2 | 54 | 2011 | Langevin, 2012 |
| China | 367 | 3.1 | 55.8 | 98 | 2008 | World Bank, 2012 |
| Ethiopia | 25 | 3.1 | 61 | 67 | 2005 | Garcia & Rajkumar, 2008 |
| India | 61 | 1.3 | 32.2 | 70 | 2014 | MoH, 2015 |
| Indonesia | 107 | 1.2 | 39 | 67 | 2008 | World Bank, 2008 |
| Mexico | 664 | 3.2 | 51.7 | 44 | 2011 | Ministry of Finance, 2011 |
| Nigeria | 115 | 1.1 | 27.6 | 64 | 2005 | Olaniyan & Lawanson, 2010 |
| South Africa | 593 | 4.3 | 48.4 | 81 | 2005 | World Bank, 2012 |

Source: Glassman and Sakuma (2014)

**PART B: Defining the Intervention Group**

The intervention group refers to the 14 most marginalized counties while the remaining 33 counties were the comparison group. The 33 counties were also the non-marginalized counties.

The intervention counties received additional resources from the national government. The transfer of additional resources to the comparison counties was based on explicit and transparent criteria. The intervention counties received these additional resources through an equalization fund (EF). The EF, established through article 204 of the 2010 Constitution, was created with the aim of addressing marginalization (and relatedly, an uneven development) across Kenya. As stated in the Constitution, the FE was created to help ensure “basic services including water, roads, health facilities and electricity to marginalized areas to the extent necessary to bring the quality of those services in those areas to the level generally enjoyed by the rest of the nation” (*See part C of this appendix for what the constitution 2010 says about the equalization fund).*

Determination on marginalization was initially done based on the recommendation made by the Commission on Revenue Allocation (CRA). The CRA recommendation was based on a formula that combined quantitative and qualitative evidence on the overall development of the counties. More specifically, the formula had three components: (a) County Development Index (CDI), (b) findings from a county-level survey that relied on direct public participation, and (c) findings based on an analysis of historical injustices. The CDI was based on indicators on poverty, infrastructure, education, and health. The county-level survey, which allowed county residents to provide their inputs on the level of marginalization present in their county, solicited qualitative information from a diverse set of stakeholders. The final list for 14 most marginalized counties was determined after considering counties’ progress on all three components. *(See parts D and E of this appendix on the details of county rankings, the methodology behind the ranking, and the final list of counties).* Although the determination of counties in two categories was finalized earlier, it became effective at the same time as decentralization became effective in July 2013 following elections for local governments in March 2013.

**PART C: Equalisation Fund**

Part A: Constitutional mandate based (verbatim) on article 204 of the 2010 Constitution:

1. There is established an Equalisation Fund into which shall be paid one half per cent of all the revenue collected by the national government each year calculated on the basis of the most recent audited accounts of revenue received, as approved by the National Assembly.
2. The national government shall use the Equalisation Fund only to provide basic services including water, roads, health facilities and electricity to **marginalised areas** to the extent necessary to bring the quality of those services in those areas to the level generally enjoyed by the rest of the nation, so far as possible.
3. The national government may use the Equalisation Fund—
4. only to the extent that the expenditure of those funds has been approved in an Appropriation Bill enacted by Parliament; and
5. either directly, or indirectly through conditional grants to counties in which marginalised communities exist.
6. The Commission on Revenue Allocation shall be consulted, and its recommendations considered before Parliament passes any Bill appropriating money out of the Equalisation Fund.
7. Any unexpended money in the Equalisation Fund at the end of a particular financial year shall remain in that Fund for use in accordance with clauses (2) and (3) during any subsequent financial year.
8. This Article lapses twenty years after the effective date, subject to clause (7).
9. Parliament may enact legislation suspending the effect of clause (6) for a further fixed period of years, subject to clause (8).
10. Legislation under clause (7) shall be supported by more than half of all the members of the National Assembly, and more than half of all the county delegations in the Senate.
11. Money shall not be withdrawn from the Equalisation Fund unless the Controller of Budget has approved the withdrawal.

**PART D: Methodology for the Identification of Marginalized Counties**

Identification of marginalized counties was based on the following three criteria:

- 1. *County development index (CDI)*
  2. *County survey*
  3. *An analysis of historical and legislated injustices*

***1. County Development Index***

CDI was a quantitative measure designed to capture the level and quality of basic services

The process of developing the CDI entailed: indicators selection, weight attribution, county ranking and threshold determination.

- 1. *Indicators selection*

CDI indicators were selected based on the following 4 dimensions:

- - - Poverty
    - Infrastructure
    - Health
    - Education
  1. *Weights*

The assignment of weights to the indicators was a function of:

- Constitutional stipulations
- Commission on Revenue Allocation (CRA) judgement

Table below details dimensions, indicators and corresponding weights assigned:

**Table A2**: Indicators Used in County Development Index

| **Dimension** | **Indicator** | **Indicator-level Weight** | **Total Weight** |
| --- | --- | --- | --- |
| Poverty | Poverty gap | 16 | 16 |
| Infrastructure | Roads | 9.33 | 28 |
|  | Electricity | 9.33 |  |
|  | Water | 9.33 |  |
| Health | Immunization | 9.33 | 28 |
|  | Sanitation | 9.33 |  |
|  | Deliveries in health facilities | 9.33 |  |
| Education | Literacy | 14 | 28 |
|  | Secondary education | 14 |  |

1. ***County survey***

- conducted by the Commission for Revenue Allocation (CRA)
- a national survey carried out in June 2012
- 150 selected participants per county
- participants included diverse stakeholders including faith-based organizations, youth and women’s groups, government officers, media, unions, and others.

1. ***Analysis of historical and legislated injustices***

- A qualitative analysis meant to provide additional context and nuance to CDI

**PART E: Classification of Counties**

| **County** | **CDI** | **Survey** | **Injustice Analysis** | **Marginalized?** |
| --- | --- | --- | --- | --- |
| TURKANA | 0.27 | 1 | 27.72 | Y |
| MANDERA | 0.31 | 1 | 8.35 | Y |
| WAJIR | 0.33 | 1 | 9.45 | Y |
| MARSABIT | 0.37 | 1 | 10.71 | Y |
| SAMBURU | 0.38 | 1 | 3.94 | Y |
| WEST POKOT | 0.38 | 1 | 5.2 | Y |
| TANA RIVER | 0.39 | 1 | 5.51 | Y |
| NAROK | 0.44 | 1 | 0.63 | Y |
| KWALE | 0.45 | 1 | 1.42 | Y |
| GARISSA | 0.47 | 1 | 1.73 | Y |
| KILIFI | 0.5 | 1 | 2.52 | Y |
| TAITA TAVETA | 0.51 | 1 | 1.42 | Y |
| ISIOLO | 0.52 | 1 | 2.2 | Y |
| LAMU | 0.56 | 1 | 9.13 | Y |
| BARINGO | 0.44 | 0 | 0.94 | N |
| KITUI | 0.46 | 0 | 1.42 | N |
| HOMA BAY | 0.47 | 0 | 0.16 | N |
| THARAKA NITHI | 0.48 | 0 | 0.79 | N |
| TRANS NZOIA | 0.49 | 0 | 0.79 | N |
| BUSIA | 0.51 | 0 | 0.63 | N |
| BOMET | 0.51 | 0 | 0 | N |
| MIGORI | 0.52 | 0 | 0.47 | N |
| KAJIADO | 0.53 | 1 | 0.47 | N |
| KISUMU | 0.53 | 0 | 0.16 | N |
| ELGEYO MARAKWET | 0.54 | 0 | 0.63 | N |
| MACHAKOS | 0.54 | 0 | 0.47 | N |
| MAKUENI | 0.54 | 0 | 0.47 | N |
| SIAYA | 0.55 | 0 | 0.31 | N |
| NANDI | 0.55 | 0 | 0.16 | N |
| MERU | 0.55 | 0 | 0 | N |
| BUNGOMA | 0.55 | 0 | 0.31 | N |
| LAIKIPIA | 0.57 | 0 | 0.63 | N |
| VIHIGA | 0.57 | 0 | 0 | N |
| KAKAMEGA | 0.57 | 0 | 0.31 | N |
| KERICHO | 0.57 | 0 | 0 | N |
| EMBU | 0.57 | 0 | 0 | N |
| MURANGA | 0.57 | 0 | 0 | N |
| NYANDARUA | 0.58 | 0 | 0.31 | N |
| KIRINYAGA | 0.6 | 0 | 0 | N |
| NAKURU | 0.6 | 0 | 0 | N |
| KISII | 0.61 | 0 | 0.16 | N |
| NYAMIRA | 0.62 | 0 | 0 | N |
| UASIN GISHU | 0.62 | 0 | 0 | N |
| NYERI | 0.64 | 0 | 0.31 | N |
| MOMBASA | 0.67 | 0 | 0.16 | N |
| KIAMBU | 0.68 | 0 | 0 | N |
| NAIROBI CITY | 0.77 | 0 | 0 | N |

Notes: CDI= county development index; Y = yes, N = No

**PART F: Difference-in-Differences**

**F.1: The Canonical DID**

The canonical difference-in-differences (DID) design assumes two periods (pre- and post-) for the two groups: intervention group and the comparison group. It can be represented by the equation below:

Y_it_ = β_0_ + β_1_ₓIntervention_i_ + β_2_ₓPost_t_ + β_3_ₓIntervention_i_*Post_t_ + ε_it_ ---------- [equation 1]

where *Y_it_* represents outcome (HIV incidence or diarrhea incidence) for county *i* in year *t,* *intervention_i_* represents the intervention status for county *i* and is equal to 1 if intervened (or marginalized) county, *post_t_* is equal to 1 if the year is 2013 or after and 0 if prior to 2013, and ε represents the error term. The coefficient of interest is β_3_, which indicates the causal impact of the intervention, i.e., the difference in outcome between intervention and comparison counties after the intervention relative to the difference in the outcome before the intervention.

**F.2: Event Study Design Based DID Set Up**

This model is an expanded (and therefore, more general) form of the canonical DiD model, which included interactions between the intervention and each time points (i.e., year) during the study window. An equation corresponding to this model is shown below:

Y_it_ = σ_0_ + σ_1_ₓIntervention_i_ + σ_2_ₓIntervention_i_*Year_t_ + €_it_ ---------- [equation 2]

Where *Year_t_* represents years between 2006 and 2019, except 2012 which was the reference year. Other terms are defined as in equation 1. The coefficient, σ_2_, represents the impact of the intervention at each point in time. Since the intervention occurred in 2013, we should not expect to see this coefficient being significant for years 2012 and before. The use of this model allowed us to estimate explicitly how the impact of the intervention changed over the course of the study period, and more specifically, after the intervention onset. Unlike in the canonical model, this model considers the mean of the outcome variable for each time/year (rather than averaging the outcome separately for the entire pre period) and the entire post period, and whether that changes because of the intervention. The key advantage of using this model is that we can link intervention effects with a specific year, thereby adding granularity to the analysis.

**F.3: Selection of the Outcomes and the Control Variables**

***Outcomes***

HIV/AIDS and diarrhea were chosen as the two health outcomes primarily because of their policy relevance in the Kenyan context. HIV/AIDS and diarrhea continue to be two of the major causes of deaths in Kenya.

***Controls/Covariates***

Generally speaking, these variables were selected as controls/covariates based on their availability and relevance. Relevance was construed in terms of the variable’s relationship with: (a) the outcome variables, or (a) both the outcome variables and the treatment variable.

GDP per capita (which represents income) was included because income is a strong determinant of health outcomes. While the role of contextual factors (i.e., county-specific factors) is key, on average, a county with a high GDP per capita is more likely to have better health outcomes than a county with a low GDP per capita. Additionally, income is related to the treatment because it was one of the several factors to determine a county’s marginalization status.

We included health expenditure per capita as a control because counties with high health expenditure per capita are more likely to have better health outcomes than counties with low health expenditure per capita. Similar to GDP per capita, this variable reflects the resources available for a county to spend. Additionally, it represents the extent to which a county prioritizes health, something that high GDP per capita alone cannot guarantee.

Maternal education (proxy for the level of education in a county) was included as a control because at an aggregate level, an educated county is more likely to have better health outcomes than a less educated county. For instance, doing behavior change communication (aimed at improving healthy habits) in an educated county may be easier than doing the same in a less educated county. Because a county’s educational status/level was one of the several inputs to ultimately determine a county’s marginalization status, maternal education is closely related to the treatment as well.

The share of the urban population, a proxy for population density, was included as a control variable because higher population density may determine the ease (or lack thereof) with which health interventions are implemented to improve health outcomes. From a sanitation perspective, for instance, densely populated counties may face more difficulties implementing sanitation improvement programs. At the same time, if HIV/AIDS is concentrated in certain parts of Kenya, it may be easier to roll out an anti-HIV/AIDS program in densely populated counties. Finally, we included the percentage of antenatal care (ANC) coverage and DTP3 vaccination among children as the two health-related controls to proxy a county’s state of health. Not only was health one of the several inputs to determine a county’s marginalization status, but it is also plausible that a county’s aggregate state/level of health shapes health outcomes.

**F.4: Results for the Test of Parallel Trends Assumption**

**Figure A1**: Pre-intervention trends for HIV incidence

**
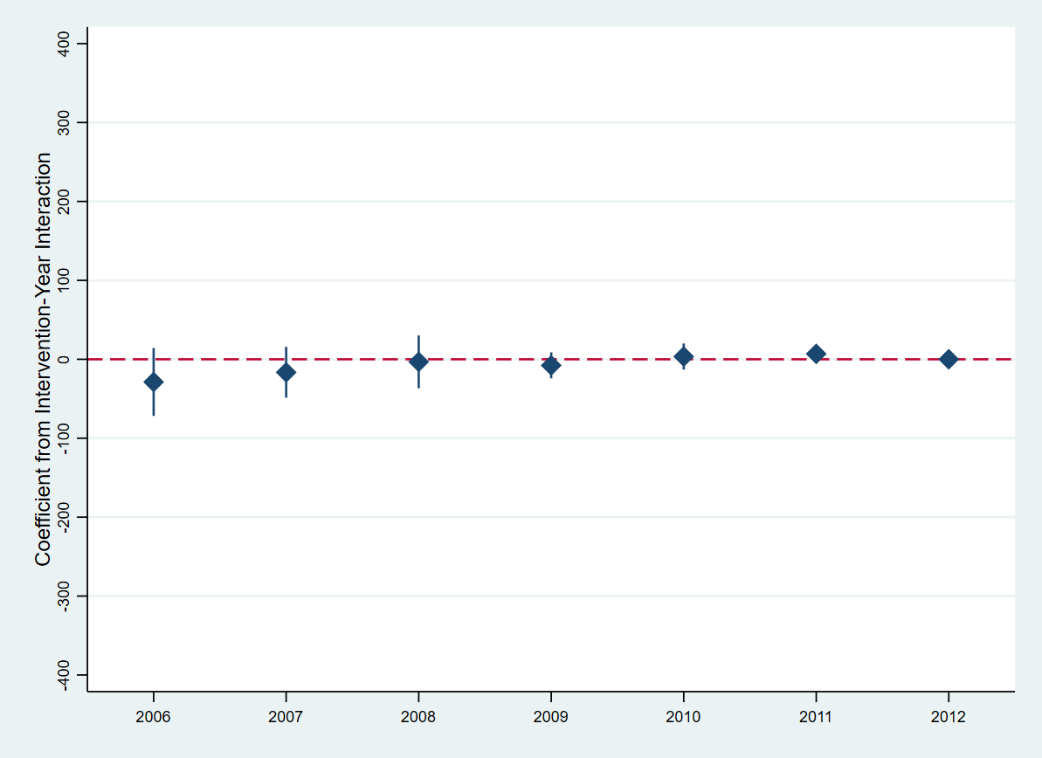
**

Figure shows, for each year between 2006 and 2013, the coefficient on the interaction between the intervention variable and the time (year) variable. The fact that the coefficients are not significantly different from zero suggests that prior to the 2013 intervention, both the intervention group and the comparison had similar trajectories for HIV incidence, the outcome variable.

**Figure A2**: Pre-intervention trends for diarrhea incidence

**
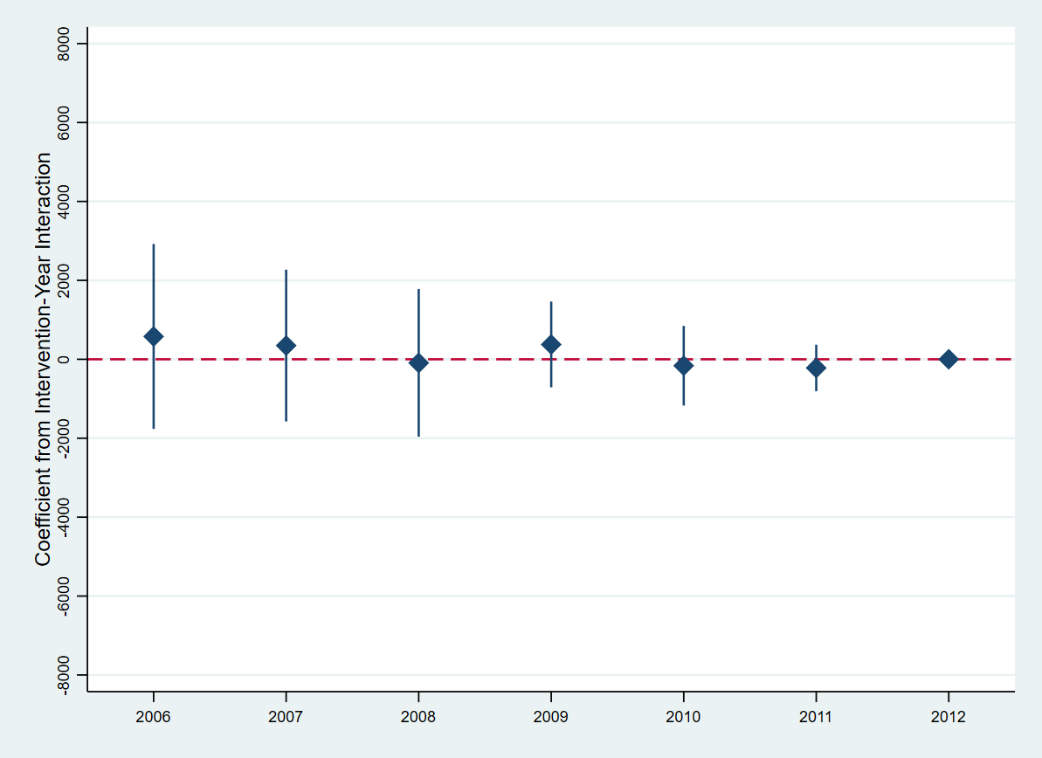
**

Figure shows, for each year between 2006 and 2013, the coefficient on the interaction between the intervention variable and the time (year) variable. The fact that the coefficients are not significantly different from zero suggests that prior to the 2013 intervention, both the intervention group and the comparison had similar trajectories for diarrhea incidence, the outcome variable.

**PART G: Sensitivity Analysis Based on Event Study Design Set-Up**

**G.1: Graphical Examination of Outcomes’ Trend During the Entire Study Period**

**Figure A3**: Impact of intervention on HIV incidence between 2006-2019

**
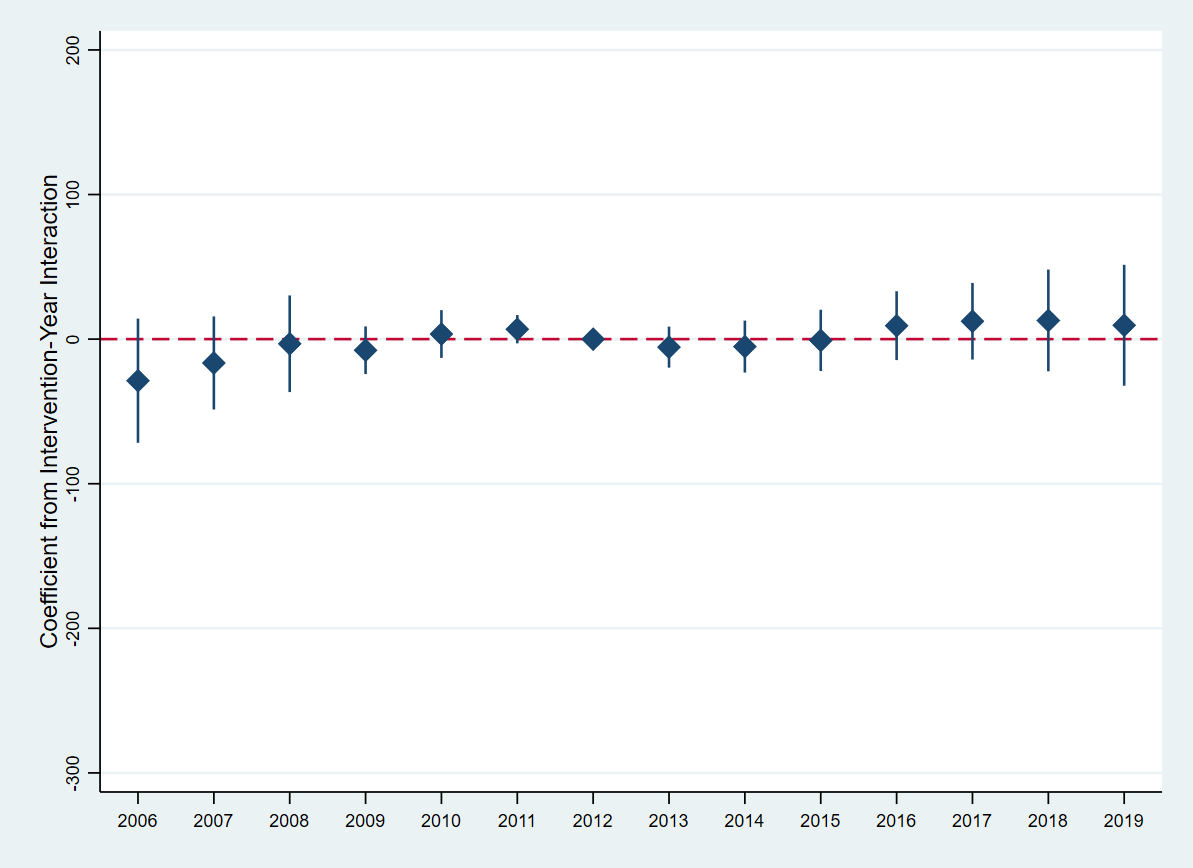
**

Figure shows, for each year between 2006 and 2019 the coefficient on the interaction between the intervention variable and the time (year) variable. The fact that the coefficients are not significantly different from zero (the dotted red line) suggests that the intervention did not have any impact on HIV incidence.

**Figure A4**: Impact of intervention on diarrhea incidence between 206-2019

**
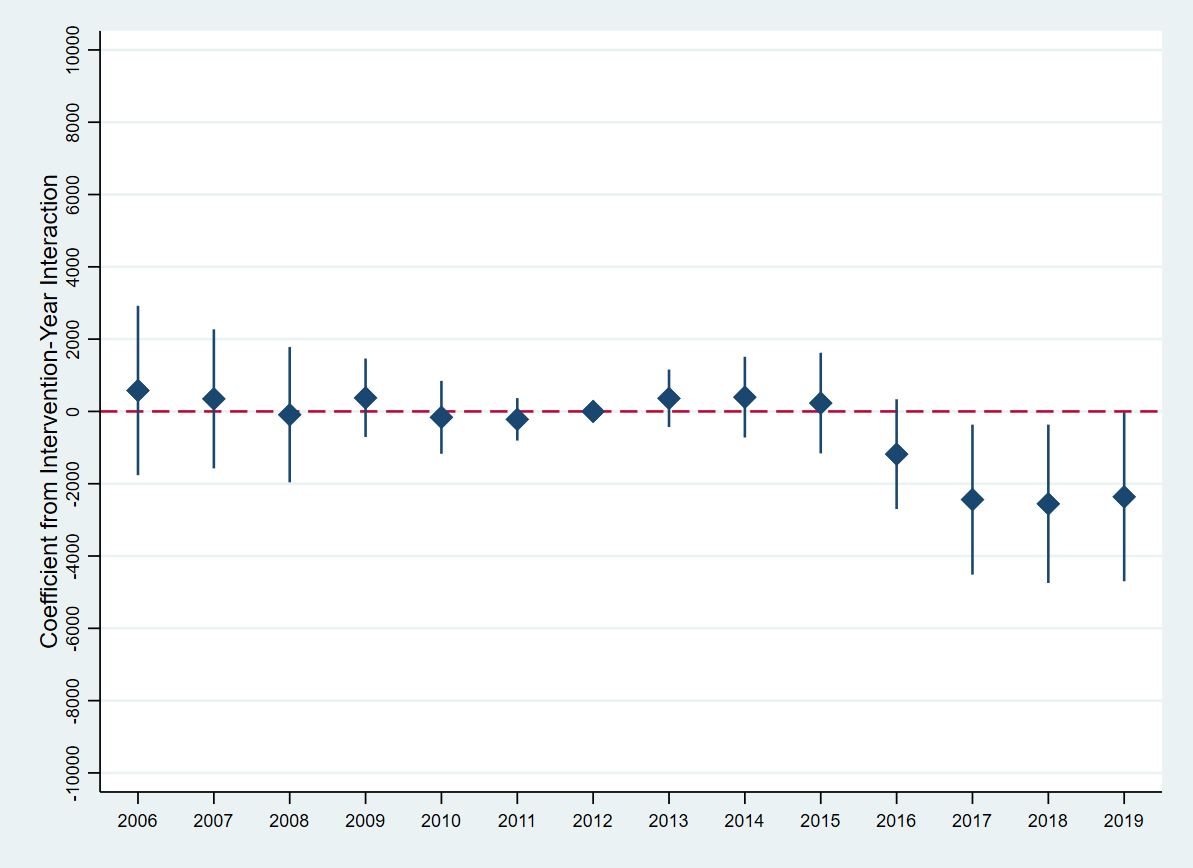
**

Figure shows, for each year between 2006 and 2019 the coefficient on the interaction between the intervention variable and the time (year) variable. The fact that the coefficients are significantly different from zero (the dotted red line) approximately after 2016 suggests that the intervention likely started having impact on diarrhea incidence starting 2016.

**Part H: Sensitivity Analysis Based on the Use of Propensity Score Matched DID Technique**

**H.1: Pre-Intervention Trends**

**Figure A5**: Pre-intervention trends for HIV incidence (using propensity score matched DID technique)


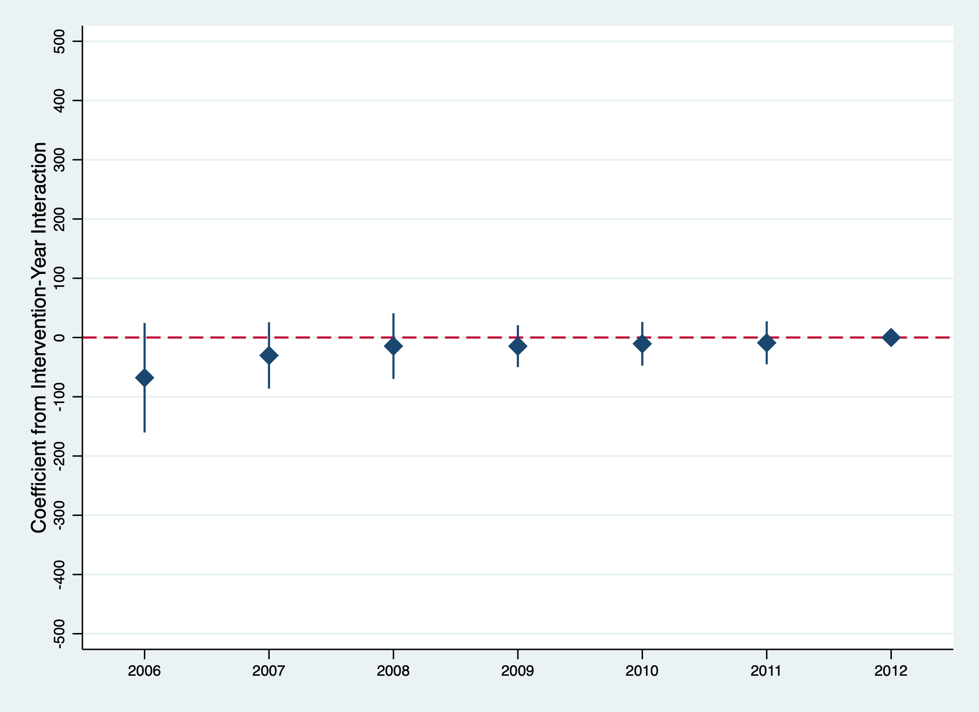


Figure shows, for each year between 2006 and 2013, the coefficient on the interaction between the intervention variable and the time (year) variable. The fact that the coefficients are not significantly different from zero suggests that prior to the 2013 intervention, both the intervention group and the comparison had similar trajectories for HIV incidence, the outcome variable.

**Figure A6**: Pre-intervention trends for diarrhea incidence (using propensity score matched DID technique)


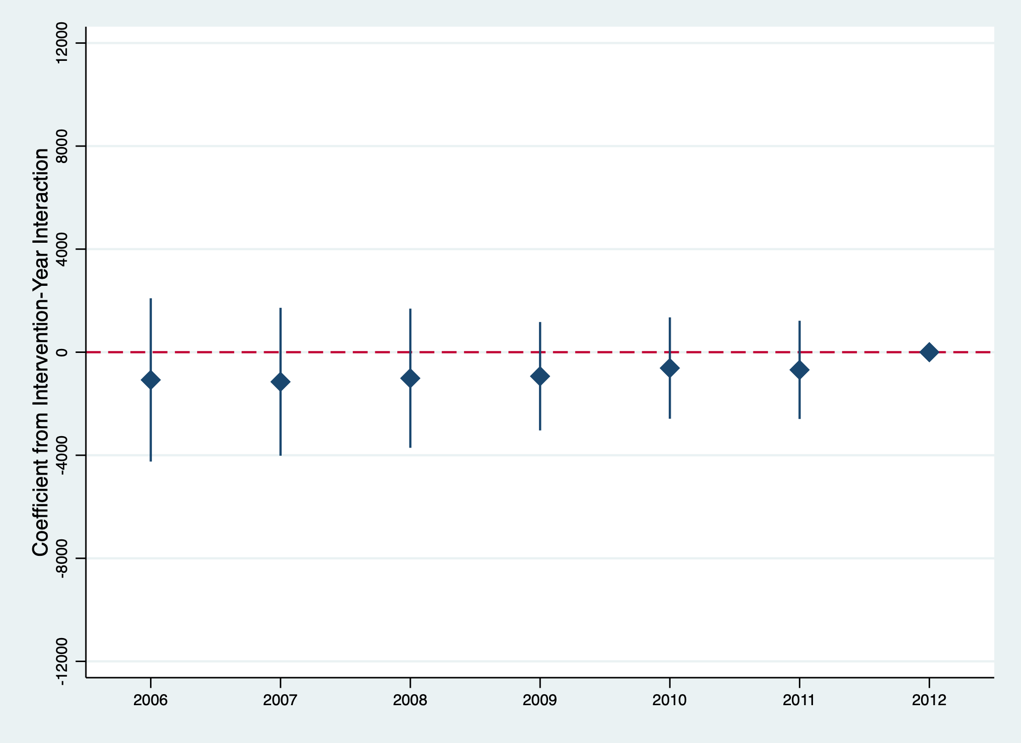


Figure shows, for each year between 2006 and 2013, the coefficient on the interaction between the intervention variable and the time (year) variable. The fact that the coefficients are not significantly different from zero suggests that prior to the 2013 intervention, both the intervention group and the comparison had similar trajectories for diarrhea incidence, the outcome variable.

**H.2: Graphical Examination of Outcomes’ Trend During the Full Study Period**

**Figure A7**: Impact of intervention on HIV incidence between 2006-2019 (using propensity score matched DID technique)


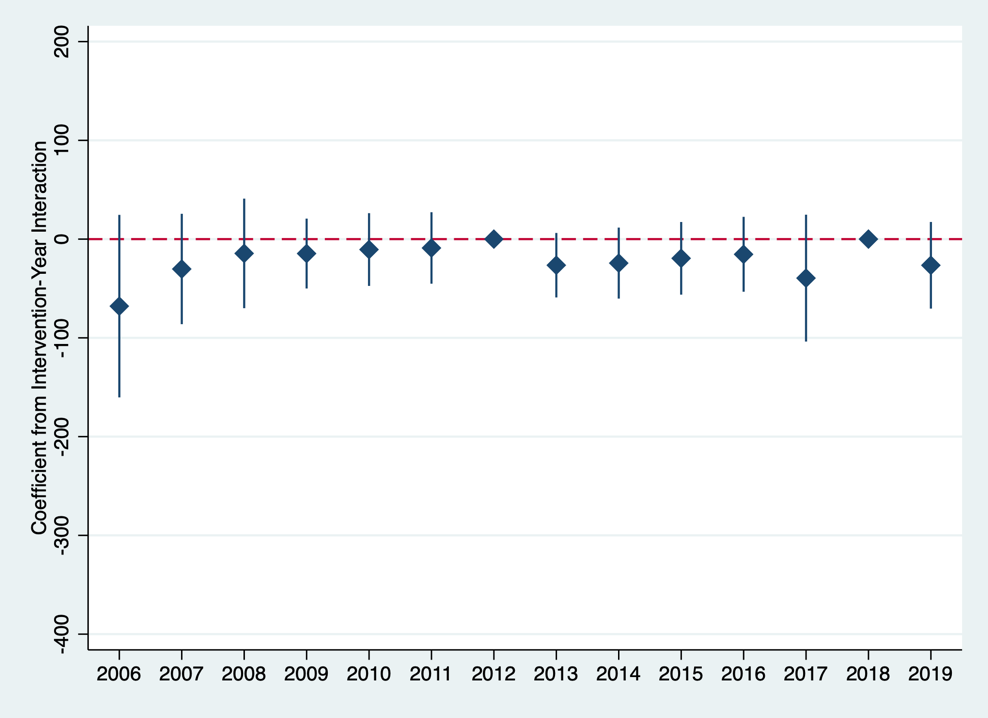


Figure shows, for each year between 2006 and 2019 the coefficient on the interaction between the intervention variable and the time (year) variable. The fact that the coefficients are not significantly different from zero (the dotted red line) suggests that the intervention did not have any impact on HIV incidence.

**Figure A8**: Impact of intervention on diarrhea incidence between 2006-2019 (using propensity score matched DID technique)


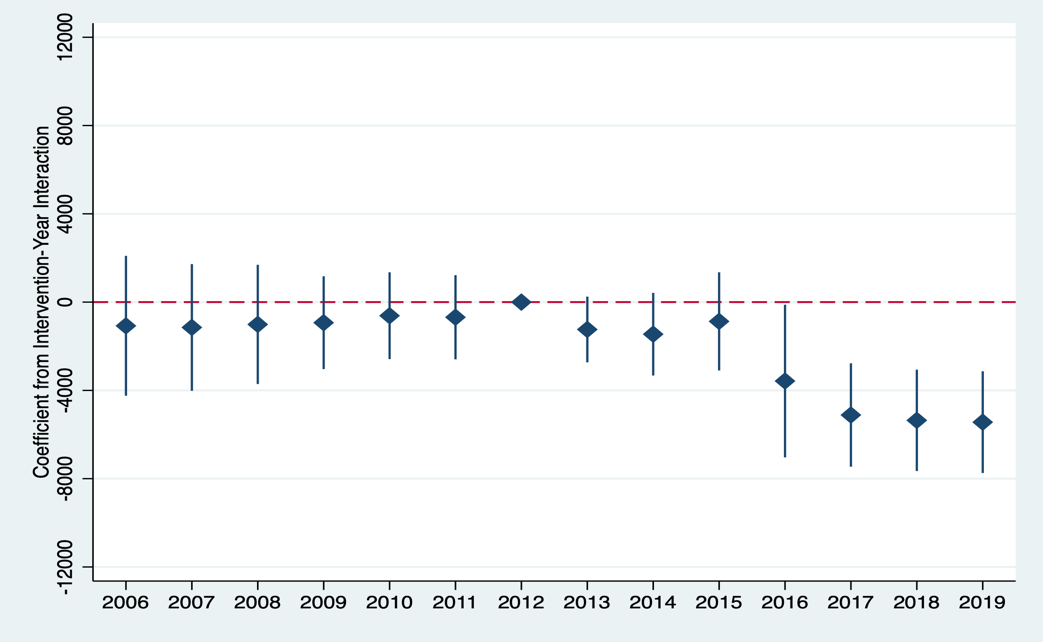


Figure shows, for each year between 2006 and 2019 the coefficient on the interaction between the intervention variable and the time (year) variable. The fact that the coefficients are significantly different from zero (the dotted red line) approximately after 2016 suggests that the intervention likely started having impact on diarrhea incidence starting 2016.

**H.3: Regression Results**

The results are based on applying propensity score matched DID technique and rely on the outcome measures meeting the conditional parallel trends assumption. The results are consistent with results from the canonical DID model.

**Table A3**: Impact of intervention on HIV incidence & diarrhea incidence (using propensity score matched DID technique)

|  | **Unadjusted** | | **Adjusted** | |
| --- | --- | --- | --- | --- |
|  | **HIV Incidence (1)** | **Diarrhea Incidence (2)** | **HIV Incidence (3)** | **Diarrhea Incidence (4)** |
| Intervention*post [DID estimate] | 9.155 | -4,254.970*** | -2.215 | -8,705.912*** |
|  | (18.017) | (1,383.643) | (23.672) | (1,487.522) |
| Constant | 249.857 | 112,763.31 | 508.405 | 109,742.09 |
|  | (9.405) | (546.160) | (82.512) | (12,168.479) |
| County-years | 242 | 262 | 242 | 262 |

Estimates in Table A2 are based on the application of propensity score matched difference-in-differences (DID) technique. Across the four models, coefficient on the interaction term is the DID estimate and represents the effect of intervention (which occurred in 2013) on the incidence of HIV and diarrhea. Incidence is expressed as the # of new cases per 100,000 population. 2006-2012 is the pre-period and 2013-2019 is the post (or intervention) period. Baseline (reference) year for all models was 2012. Adjusted models adjust for the following covariates: income, maternal education, proportion of urban population, antenatal care (ANC) coverage rate, health expenditure per capita, and DTP3 vaccination rate. N corresponds to the number of county-years. ***p<0.01, ** p<0.05, * p<0.1. Standard errors are provided in parentheses.

**H.4: Regression Results of the Placebo Test**

**Table A4**: Placebo test for the impact of intervention on HIV incidence & diarrhea incidence (using propensity score matched DID technique)

|  | **Unadjusted** | | **Adjusted** | |
| --- | --- | --- | --- | --- |
|  | **HIV Incidence (1)** | **Diarrhea Incidence (2)** | **HIV Incidence (3)** | **Diarrhea Incidence (4)** |
| Intervention*post [DID estimate] | 25.643 | 323.548 | 16.515 | 1,097.314 |
|  | (17.556) | (765.669) | (24.558) | (936.865) |
| Constant | 259.734 | 112853.927 | 602.835 | 129498.672 |
|  | (5.970) | (347.710) | (72.568) | (7,954.880) |
| County-years | 152 | 168 | 152 | 168 |

Table A3 presents results of the placebo test. The placebo test was applied in the propensity score matched DID model and relied on one key assumption. The assumption was that the intervention occurred in 2009, three years earlier than the time of the true intervention. The pre-intervention window included 2006, 2007, and 2008. Post-intervention window included 2010, 2011, and 2012. Years after 2012 were excluded from the analysis. Across the four models, coefficient on the interaction term is the DID estimate and represents the effect of intervention (which hypothetically occurred in 2009) on the incidence of HIV and diarrhea. Incidence is expressed as the # of new cases per 100,000 population. Baseline (reference) year for all models was 2009. Adjusted models adjust for the following covariates: income, maternal education, proportion of urban population, antenatal care (ANC) coverage rate, health expenditure per capita, and DTP3 vaccination rate. N corresponds to the number of county-years. ***p<0.01, ** p<0.05, * p<0.1. Standard errors are provided in parentheses.

**PART I: Sensitivity Analysis Assuming Poisson Distribution of the Outcome Measures**

**Table A5**: Difference-in-Differences Estimates [Poisson Model]

| Intervention*Post | IRR | P-value | 95% CI | |
| --- | --- | --- | --- | --- |
| HIV Incidence | 0.92 | 0.07 | 0.84 | 1.01 |
| Diarrhea Incidence | 0.95 | 0.00 | 0.93 | 0.97 |

Table shows adjusted Incidence Rate Ratio (IRR) estimates for the canonical difference-in-differences (DID) under the assumption that the outcome variable follows a Poisson distribution. Estimates for both HIV incidence and diarrhea incidence are presented in the same table here. An IRR less than 1 indicates that the intervention was associated with a decrease in the outcome variable (i.e., the incidence of HIV and diarrhea).

**Table A6**: Event-Study Design Based Regression Estimates [Poisson Model]

|  | HIV Incidence | | | |  | Diarrhea Incidence | | | |
| --- | --- | --- | --- | --- | --- | --- | --- | --- | --- |
|  | IRR | P-value | 95% CI | |  | IRR | P-value | 95% CI | |
| Intervention*2006 | 0.98 | 0.73 | 0.89 | 1.08 |  | 1.00 | 0.98 | 0.98 | 1.02 |
| Intervention*2007 | 0.98 | 0.61 | 0.90 | 1.06 |  | 1.00 | 0.95 | 0.98 | 1.02 |
| Intervention*2008 | 0.99 | 0.86 | 0.92 | 1.07 |  | 1.00 | 0.82 | 0.98 | 1.01 |
| Intervention*2009 | 1.00 | 0.93 | 0.95 | 1.05 |  | 1.00 | 0.57 | 0.99 | 1.01 |
| Intervention*2010 | 1.00 | 0.94 | 0.96 | 1.04 |  | 1.00 | 0.88 | 0.99 | 1.01 |
| Intervention*2011 | 1.00 | 0.98 | 0.97 | 1.03 |  | 1.00 | 0.57 | 0.99 | 1.00 |
| Intervention*2013 | 0.99 | 0.72 | 0.97 | 1.02 |  | 1.00 | 0.53 | 1.00 | 1.01 |
| Intervention*2014 | 1.00 | 0.86 | 0.96 | 1.04 |  | 1.00 | 0.67 | 0.99 | 1.01 |
| Intervention*2015 | 0.99 | 0.73 | 0.94 | 1.05 |  | 1.00 | 0.96 | 0.99 | 1.01 |
| Intervention*2016 | 1.00 | 0.89 | 0.93 | 1.07 |  | 0.99 | 0.05 | 0.97 | 1.00 |
| Intervention*2017 | 1.00 | 0.99 | 0.92 | 1.08 |  | 0.97 | 0.01 | 0.96 | 0.99 |
| Intervention*2018 | 0.96 | 0.35 | 0.87 | 1.05 |  | 0.97 | 0.00 | 0.95 | 0.99 |
| Intervention*2019 | 0.92 | 0.13 | 0.83 | 1.02 |  | 0.97 | 0.01 | 0.95 | 0.99 |

Table shows adjusted Incidence Rate Ratio (IRR) estimates for the event-study type of design, under the assumption that the outcome variable follows a Poisson distribution. Estimates for both HIV incidence and diarrhea incidence are presented in the same table here. An IRR less than 1 indicates that the intervention was associated with a decrease in the outcome variable (i.e., the incidence of HIV and diarrhea).

**PART J: Sensitivity Analysis Using Prevalence Rate as the Outcome Measure**

**Table A7:** Difference-in-Differences Estimates [outcome = prevalence]

|  | HIV Prevalence (1) | Diarrhea Prevalence (2) |
| --- | --- | --- |
| Intervention*Post | -30.944 | -78.573*** |
|  | (93.727) | (20.359) |
| Constant | 7,399.976*** | 1,426.352*** |
|  | (806.087) | (194.517) |
| County-years | 658 | 658 |

Table 2 presents impact of difference-in-difference (DID) estimates of the impact of intervention on HIV prevalence and diarrhea prevalence. Adjusted results control for income, maternal education, proportion of urban population, antenatal care (ANC) coverage rate, health expenditure per capita, and DTP3 vaccination rate. All models include year and county fixed effects. 2006-2012 is the pre-period and 2013-2019 is the post (or intervention) period. Baseline (reference) year for all models is 2012. ***p<0.01, ** p<0.05, * p<0.1.

**Table A8:** Event-Study Design Based Estimates [Outcome = Prevalence Rate]

|  | HIV Prevalence (1) | Diarrhea Prevalence (2) |
| --- | --- | --- |
| 2012 (reference year) |  |  |
| Intervention*2006 | -114.723 | 11.215 |
|  | (115.854) | (21.567) |
| Intervention*2007 | -63.038 | 5.921 |
|  | (92.211) | (17.759) |
| Intervention*2008 | -26.080 | -1.672 |
|  | (81.278) | (17.337) |
| Intervention*2009 | -26.797 | 6.156 |
|  | (49.580) | (10.108) |
| Intervention*2010 | -3.563 | -3.603 |
|  | (38.818) | (9.367) |
| Intervention*2011 | 4.866 | -4.559 |
|  | (21.874) | (5.488) |
| Intervention*2013 | -11.983 | 6.530 |
|  | (28.482) | (7.351) |
| Intervention*2014 | -7.099 | 7.783 |
|  | (41.122) | (10.252) |
| Intervention*2015 | 9.114 | 5.182 |
|  | (54.176) | (12.708) |
| Intervention*2016 | 27.992 | -18.027 |
|  | (62.982) | (13.647) |
| Intervention*2017 | 42.978 | -37.491** |
|  | (77.028) | (18.346) |
| Intervention*2018 | 51.954 | -39.772** |
|  | (89.665) | (19.549) |
| Intervention*2019 | 67.591 | -36.083* |
|  | (107.704) | (20.945) |
| Constant | 5,152.283*** | 1,406.969*** |
|  | (1,650.066) | (314.091) |
| County-years | 658 | 658 |

Table shows coefficient estimates for the effect of intervention (which occurred in 2013) on the prevalence of HIV and diarrhea over time. 2006-2012 is the pre-period and 2013-2019 is the post (or intervention) period. Baseline (reference) year for all models is 2012. Adjusted models in the table adjust for income, maternal education, proportion of urban population, antenatal care (ANC) coverage rate, health expenditure per capita, and DTP3 vaccination rate. N corresponds to the number of county-years. ***p<0.01, ** p<0.05, * p<0.1.

**Part K: Tracking Sectoral Spending, 2014-2019**

**Figure A5**: Social sector spending, 2014-2019

**
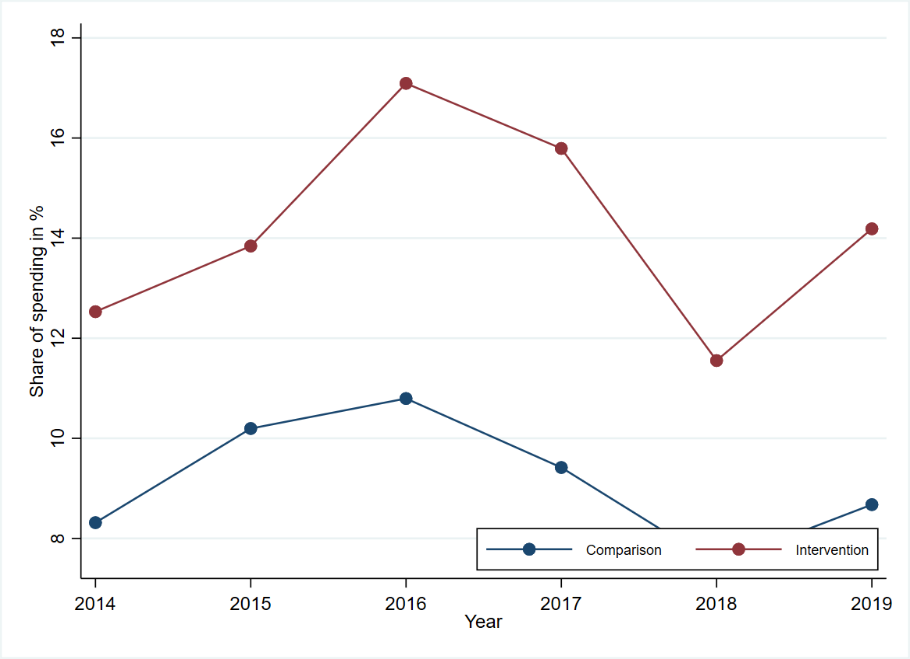
**

Social sector= health, education, and water, sanitation, and hygiene (WASH)

**Figure A6**: Health sector spending, 2014-2019

**
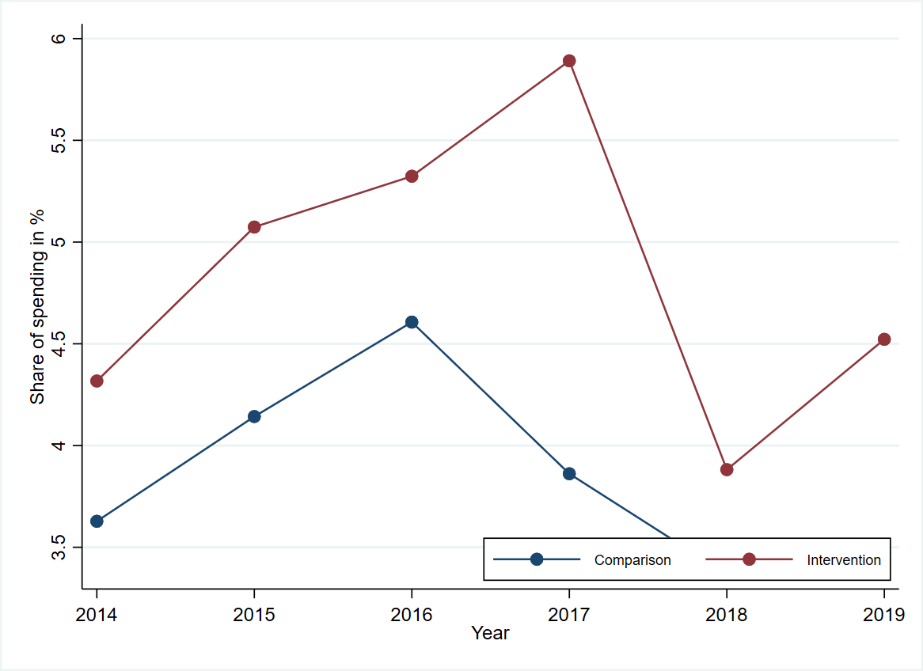
**

**Figure A7**: WASH Spending, 2014-2019

**
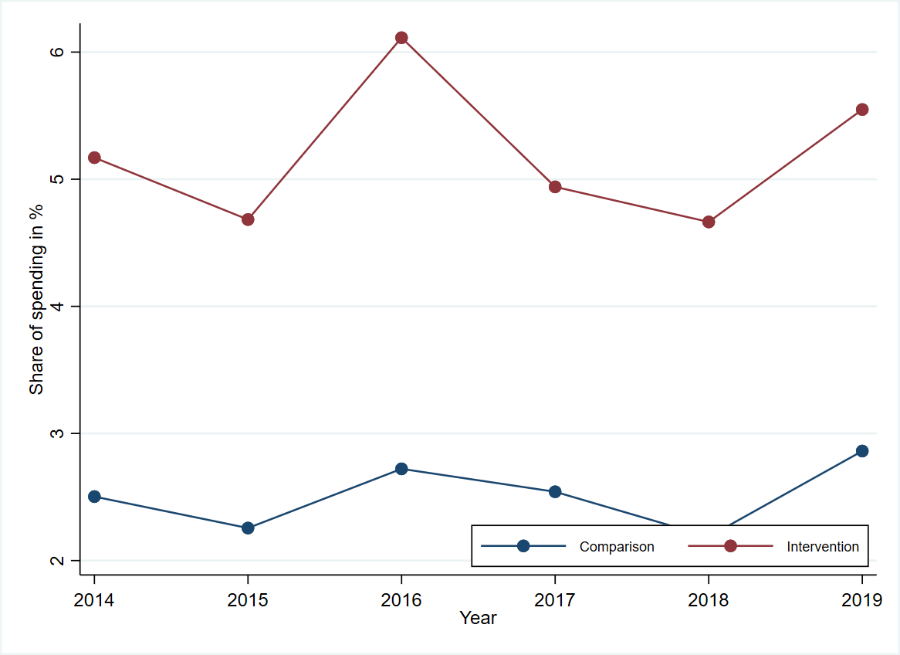
**

WASH = Water, Sanitation, and Hygiene
